# Fatigue-induced changes in hamstrings’ active muscle stiffness: effect of contraction type and implications for strain injuries

**DOI:** 10.1007/s00421-022-05104-0

**Published:** 2022-12-10

**Authors:** Pavlos E. Evangelidis, Xiyao Shan, Shun Otsuka, Chi Yang, Takaki Yamagishi, Yasuo Kawakami

**Affiliations:** 1 Faculty of Sport Sciences, Waseda University, Tokyo, Japan; 2 Japan Society for the Promotion of Science, Tokyo, Japan; 3 Department of Anatomy, Aichi Medical University, Tokyo, Japan; 4 Graduate School of Sport Sciences, Waseda University, Tokyo, Japan

**Keywords:** Hamstrings, Fatigue, Muscle stiffness, Eccentric contractions, Shear wave elastography, Strain injuries

## Abstract

**Purpose:**

Hamstring strain injuries may occur due to differential fatigue and compromised mechanical properties among the hamstring muscles. We examined (1) the effect of fatigue on hamstrings active muscle stiffness, and (2) whether contraction type affects active muscle stiffness changes during a submaximal fatiguing task.

**Methods:**

Nine healthy males completed 99 submaximal knee flexions in isometric (ISO), concentric (CON), and eccentric (ECC) conditions. We measured the knee flexor maximal voluntary torque (MVT) (pre/post), shear wave velocity (SWV) during contraction and transverse relaxation times (T2) (pre/post) in biceps femoris long head (BFlh), semitendinosus (ST), and semimembranosus (SM) muscles.

**Results:**

MVT decreased substantially after all conditions (− 18.4 to − 33.6%). The average relative torque sustained during the task was lower in CON than ISO and ECC, but absolute torque was similar. SWV interindividual responses were highly variable across muscles and contraction types. On average, BFlh SWV tended to increase in ISO (0.4 m/s, 4.5%,* p* = 0.064) but decreased in ECC condition (− 0.8 m/s, − 7.7%,* p* < 0.01). ST SWV decreased in CON (− 1.1 m/s, − 9.0%,* p* < 0.01), while it remained unchanged in ISO and ECC. SM SWV decreased in CON (− 0.8 m/s, − 8.1%,* p* < 0.01), but it was unaffected in ISO and variable in ECC.

**Conclusion:**

Fatigue has a differential effect on the mechanical properties of the constituent hamstring muscles, as measured with shear wave elastography, depending upon contraction type. We found preliminary evidence that BFlh is more fatigued than ST or SM during eccentric contractions, which may explain its susceptibility to strain injuries.

**Supplementary Information:**

The online version contains supplementary material available at 10.1007/s00421-022-05104-0.

## Introduction

Fatigue [as determined by performance fatigability—the decline in muscle activation and contractile function (Enoka and Duchateau 2016)] has been long considered a risk factor for hamstring strain injuries (HSIs) (Worrell 1994; Opar et al. 2012); however, supporting evidence is largely indirect. Epidemiological data show that HSIs are more likely to occur later in a soccer or rugby match, presumably due to increasing fatigue (Woods et al. 2004; Brooks et al. 2006; Ekstrand et al. 2011), while experimental studies report reduced knee flexor maximal torque and rate of force development (Greig 2008; Greco et al. 2013; Marshall et al. 2014), hamstrings muscle electrical activity (Marshall et al. 2014), and altered sprinting kinematics (Small et al. 2009). Although these joint- and whole-body level observations allude towards possible mechanisms, a more direct insight into how fatigue predisposes hamstrings, and biceps femoris long head (BFlh) in particular, to strain injury is necessary.

A potential mechanism is that of selective recruitment and fatigue within the hamstring muscle group, that could disturb the load distribution among its constituent muscles (i.e., the force generated/ sustained by each muscle) and create excessive shearing stresses and strains. A fatigued muscle has a compromised capacity to actively exert force and resist mechanical strains, becoming susceptible at loads and strain magnitudes that could previously bear successfully (Mair et al. 1996). Although the distribution of actual forces within a muscle group cannot be determined in vivo, inferences can be drawn from the measurement of muscle electrical activity (electromyography, EMG) (Akima et al. 2002), metabolic activity (functional MRI) (Schuermans et al. 2014), and active stiffness (shear wave elastography, SWE) (Bouillard et al. 2014; Evangelidis et al. 2021). Variable hamstrings’ load distribution patterns have been reported depending upon whether the examined task is hip- or knee-dominant, the type of contraction, and range of motion (Kubota et al. 2007; Ono et al. 2010, 2011; Bourne et al. 2017; Hegyi et al. 2019; Boyer et al. 2021). When all three biarticular hamstrings were examined in a knee flexion task, semitendinosus (ST) often exhibited greater neural activation, metabolic activity, and active stiffness than BFlh and semimembranosus (SM), suggesting a higher load borne by ST (Kubota et al. 2007; Schuermans et al. 2014; Bourne et al. 2017; Evangelidis et al. 2021), although different patterns have also been observed (Avrillon et al. 2018; Boyer et al. 2021). However, little is known about how fatigue affects this pattern. Timmins et al. (2014) found that, following repeated sprints, muscle electrical activity (measured with surface EMG) decreased in BF but not in medial hamstrings (ST and SM) during maximal eccentric knee flexions, while similar responses were also seen during a simulated soccer match (Marshall et al. 2014). In contrast, two recent SWE studies reported a decrease in ST shear elastic modulus (i.e., index of tissue stiffness, directly related to muscle force), but no change in BFlh during a low-intensity sustained isometric contraction in healthy individuals (Mendes et al. 2020) and previously injured elite footballers (Freitas et al. 2021), suggesting greater fatigue in ST. These findings support the concept of differential fatigue within the hamstrings; however, they point to different muscle being affected. Fatigue is task-dependent (Enoka and Duchateau 2008), and the disparity in the results of the aforementioned studies may be due to the different tasks and contraction types examined. Hamstrings’ action during sprinting incorporates a large eccentric component that causes characteristic changes in muscle structure and function (Clarkson and Hubal 2002) and, more importantly, it is considered responsible for the HSIs (Heiderscheit et al. 2005; Schache et al. 2009). Thus, any fatigue-induced change in hamstrings load distribution may depend on contraction type; however, this has not been investigated.

In this study, we combined ultrasound SWE and MRI to examine the effect of fatigue on hamstrings muscle stiffness and load bearing pattern during an isometric, concentric, and eccentric task. Both of these methods have been previously employed to examine the load bearing pattern in hamstrings (Schuermans et al. 2014; Freitas et al. 2021) and offer complementary advantages; SWE provides real-time measurements during contraction, while MRI has greater spatial resolution. Based on previous evidence, we hypothesized that fatigue would have a contraction-type-specific effect on hamstrings load bearing pattern. Specifically, we hypothesized that BFlh would exhibit greater fatigue and lower stiffness compared to ST during the eccentric task, while ST stiffness would be more compromised in the isometric than the eccentric condition.

## Materials and methods

### Participants

Ten young, healthy males were originally recruited [also examined in Evangelidis et al. (2021)]; however, one participant dropped out for personal reasons before completing the study [n = 9, age 21.8 ± 4.2 years, height 172.8 ± 5.4 cm; body mass 67.1 ± 12.1 kg and iPAQ score 1768 ± 1453 MET-minutes/week (mean ± SD)]. Participants provided written informed consent and completed health screen and physical activity questionnaires (iPAQ short version) (Craig et al. 2003) prior to any measurements. Exclusion criteria were a history of hamstring and knee joint injuries. The study followed the principles of the Declaration of Helsinki, and it was approved by the Waseda University Ethics Committee on Human Research [2017–094].

### Overview

**Fig. 1:**
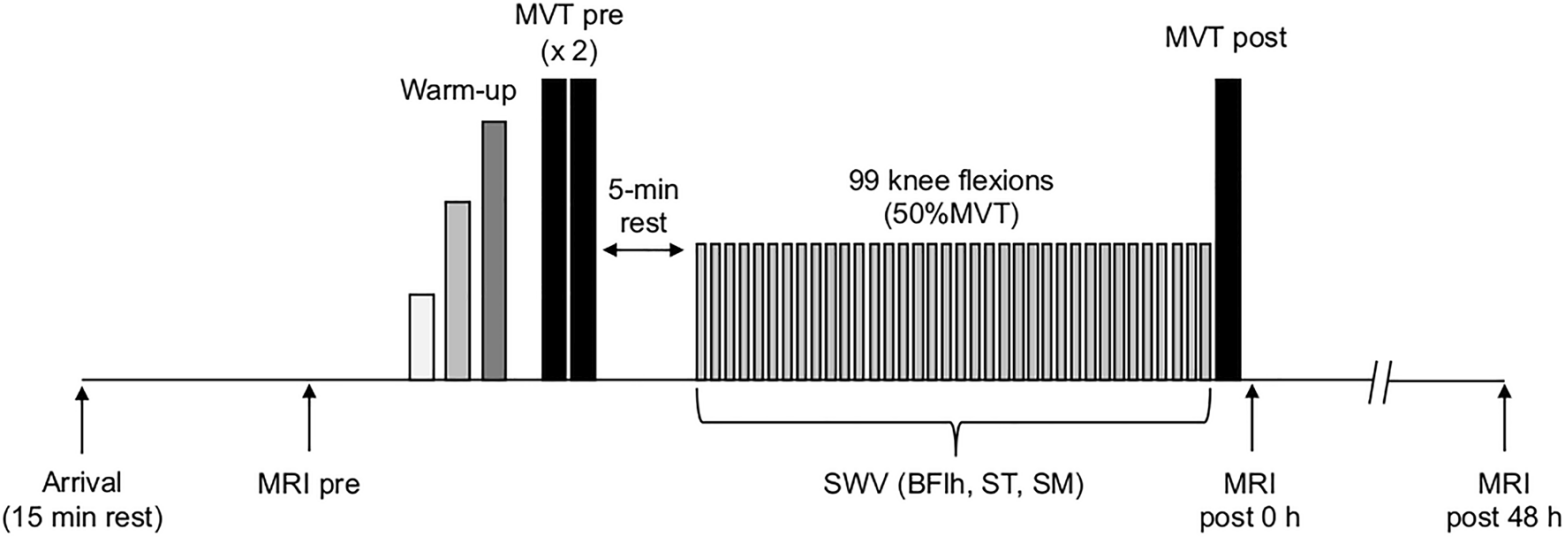
Schematic of a main session along with its complementary session 48 h later. All main sessions were identical apart from the contraction mode (isometric, concentric, or eccentric). At least 7 days were allowed between the main sessions, and a minimum of 14 days after the completion of the eccentric condition. All main sessions were randomised

Participants visited the lab at a consistent time of the day, having avoided any strenuous activity for at least 48 h before each session. In the familiarisation session, participants were thoroughly acquainted with the experimental procedures and their anthropometric data were collected. In the main sessions, upon arrival to the lab, participants rested for 15 min before the baseline MRI scan (Fig. 1). Then, they lay prone on an isokinetic dynamometer, and passive torque was recorded. After the completion of a standardised warm-up with submaximal knee flexions (3 × 50%, 2 × 70%, 1 × 90% of maximal effort), participants performed two maximal voluntary contractions (MVC) with 1 min rest. After a 5 min rest, participants completed an identical fatiguing task of 99 submaximal knee flexions (detailed below) in either isometric (ISO), concentric (CON) or eccentric (ECC) contraction mode. Active muscle shear wave velocity (SWV) was measured in BFlh, ST and SM in an alternating fashion (33 recordings per muscle). Immediately after, participants performed another MVC, and they were quickly wheeled to the MRI scanner for the post-measurement (post 0 h). A final scan was taken 48 h later. The order of the SWV measurements was randomised and counterbalanced across participants, but it remained consistent for each participant across conditions. The order of the main sessions was randomised and counterbalanced. Seven days were allowed after the ISO and CON sessions and 2 weeks after the ECC session to avoid residual fatigue or muscle soreness in the subsequent session. All measurements were performed on the right leg.

### Isokinetic dynamometry

As described previously (Evangelidis et al. 2021), participants lay prone on the isokinetic dynamometer (Con-Trex MJ, CMV AG, Dübendorf, Switzerland) at 30° of hip flexion (0° full extension). Inelastic straps across the pelvis, torso, and above the knee of the right leg were used to minimise any extraneous movements. An additional strap was placed over the extended non-involved leg. The lateral femoral condyle of the right leg was carefully aligned with the dynamometer rotational axis. The dynamometer’s shin brace was placed posterior to the shank ~ 2 cm above the medial malleolus and the shank was tightly secured to the dynamometer lever arm. For the dynamic conditions, the range of motion was 0–40°, while for the ISO, the crank was set at 30° from full extension. These angles were selected as they are close to knee flexors’ expected angle of peak torque (Ayala et al. 2013). Passive torque measured before the fatiguing task was used to correct all subsequent torque measurements. The task consisted of 99 contractions of 5 s duration at 50% maximal voluntary torque (MVT) (based on the instantaneous peak torque recorded during the MVCs), interspersed with 5 s rest. For the dynamic contractions, the angular velocity was set at 8° s^−1^. This velocity was selected to ensure that sufficient elastography data were collected during each contraction, accounting for the low temporal resolution (1 Hz) of the SWE scanner used in this study (see below). At the selected angular velocity, five shear wave measurements were taken over the examined range of motion. To match the contraction time between dynamic and isometric conditions, participants maintained the isometric contractions for 5 s. A monitor placed in front of the participants provided real-time visual feedback by displaying their torque response and a horizontal line at the target torque level, which they were instructed to match as quickly as possible. An investigator using a stopwatch provided verbal cues for the beginning and end of the contractions and standard verbal encouragement.

Torque, crank angle, and velocity signals were sampled at 2000 Hz with an A/D converter (PowerLab, ADInstruments, Australia) and filtered with a fourth-order Butterworth-type filter (cut-off frequency determined with residual analysis (Robertson et al. 2014); torque: 14 Hz, crank angle/velocity: 4 Hz). For the dynamic conditions, only data within the isovelocity phase (± 10% of the preset angular velocity) were used in further analyses. In each condition, the knee flexor MVT was determined as the highest instantaneous torque recorded from the two MVCs. Regarding the fatiguing contractions, torque data within the isovelocity phase (CON, ECC) and over the 5-s contraction (ISO) were averaged before further analysis.

### Shear wave elastography (SWE)

Muscle SWV was measured with an Aixplorer ultrasound scanner (SuperSonic Imagine, Aix-en-Provence, France) using a linear transducer (SL15-4, 4–15 Hz) and constant settings (musculoskeletal preset, smoothing 9, persistence high) as previously described (Evangelidis et al. 2021). Briefly, measurements were taken at 50% of the thigh length (greater trochanter to knee joint space). Generous amount of ultrasound gel was applied on the skin and the transducer was held firmly, perpendicular to the skin. The optimal transducer position was identified as the position where the aponeuroses and muscle fascicles were clearly visible. For the accurate replication of the transducer’s position, the skin was marked with surgical markers and B-mode ultrasound images were taken for guidance. Also, participants were provided with surgical markers, and they were instructed to maintain the marks between sessions.

**Fig. 2:**
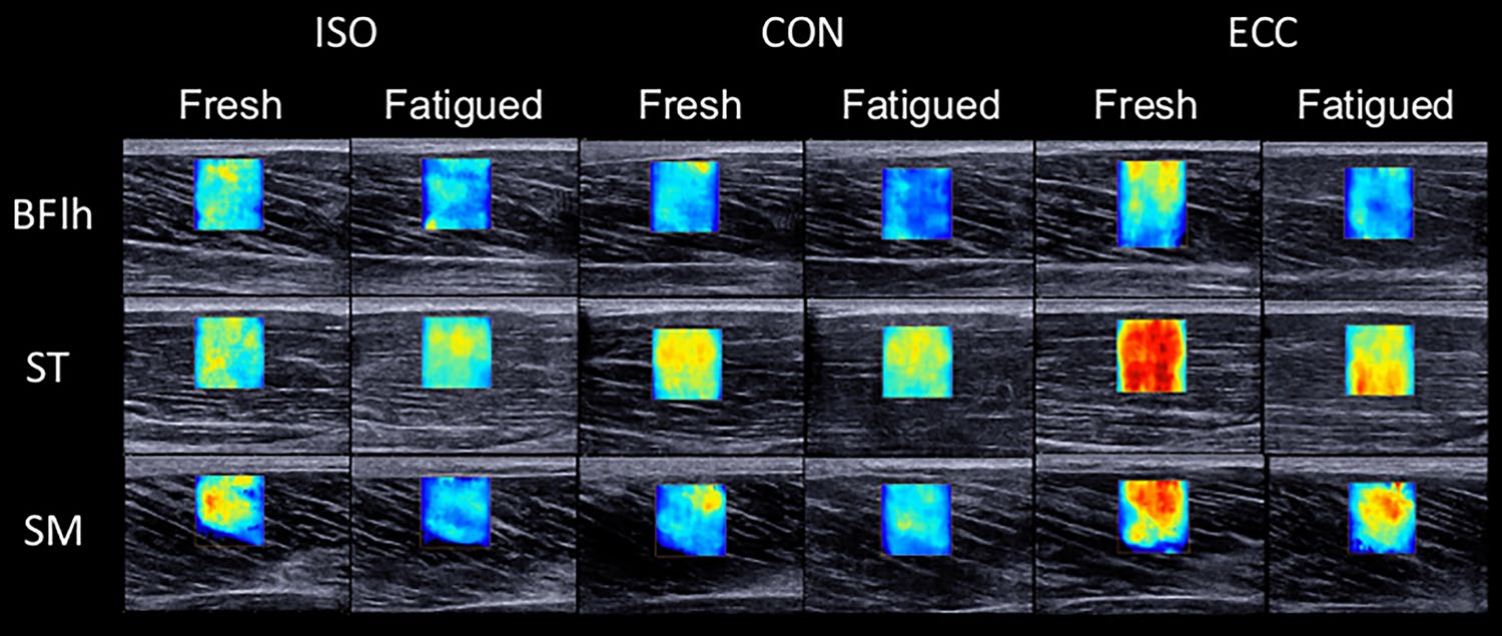
Representative shear wave elastography data during contraction at the start and end of the fatiguing task across all hamstring muscles and conditions (participant 8). Warmer colors indicate higher shear wave velocity. *ISO* isometric, *CON* concentric, *ECC* eccentric. *BFlh* biceps femoris long head, *ST* semitendinosus, *SM* semimembranosus

During the measurements, the region of interest (ROI) of the elastogram was carefully placed over the centre of the muscle belly, avoiding any non-contractile tissues both at rest and during contraction. The maximum ROI dimensions permitted by the scanner software were used. A foot pedal was used to initiate data recording and a pulse generated in PowerLab facilitated synchronisation of the elastography and dynamometry data. SWV recording began ~ 3 s prior to each contraction to allow the elastogram to stabilise and minimise artifacts. During the fatiguing task, SWV was recorded in an alternating fashion among hamstrings, for a total of 33 recordings per muscle (Fig. 2). All measurements were conducted by the same investigator.

The SWE videos were exported and analysed with a MATLAB custom script (MathWorks Inc, Natick, USA). The analysis involved drawing the largest possible rectangular ROI within the recorded elasticity map, avoiding any rejection areas and/or artifacts, and the SWV was calculated for every second. SWV values within 10% of the peak SWV (in each contraction) were averaged to obtain the representative value. Each muscle’s SWV data were averaged every three contractions, resulting in 11 final data points before further analyses.

### Magnetic resonance imaging (MRI)

Oil capsules were placed on the lateral side of the right leg at 40, 50, and 60% thigh length and participants lay supine (hip and knee joints fully extended) in a 1.5 T MRI scanner (Signa HDxt, GE). T2-weighted images (image matrix 256 × 256 mm, field of view 24 cm, repetition time 2000 ms, echo times 25, 50, 75, 100 ms, slice thickness 10 mm) were acquired with an eight-channel body array coil. Care was taken to replicate the exact position on the scan table before and after the fatiguing task. The images were analysed with Osirix software (T2 fit map plugin). First, each muscle’s ROI was manually outlined, excluding any visible connective and/or adipose tissue. Then, T2 relaxation times were calculated using the equation S_n_ = S_0_ exp^(−TEn/T2)^, where TE_n_ is echo time (*n* = 25, 50, 75 and 100 ms), S_0_ corresponds to signal intensity at 0 ms, and S_n_ is the signal intensity at TE_n_ (Maeo et al. 2018). For each muscle, the average T2 values across the three measurement sites were used for further analyses.

### Statistical analysis

Descriptive data are presented as mean ± SD. Normal distribution of the data was examined with the Shapiro–Wilk test. MVT changes were examined with a two-way repeated-measures analysis of variance (ANOVA) [factors (levels); contraction type (3) × time (2)]. Linear regressions were fitted to the individual SWV data for each muscle and contraction type and the slope of the regression line was used to calculate the overall SWV change during the task (slope × 100, ΔSWV). The regression model was also used to calculate the SWV change relative to the start of the task. To classify the absolute SWV changes, we used as a threshold the respective typical error (TE) for measurements in the same muscle, contraction mode, and intensity (50%MVT) derived from our previous study in the same cohort (Evangelidis et al. 2021). Changes equal or higher/lower than the TE were classified as increase/decrease, respectively, while changes within that range were considered as non-substantial (Bouillard et al. 2014). Analytically, these thresholds were (m/s): ISO, BFlh: 0.3, ST: 0.8, SM: 1.0; CON, BFlh: 0.6, ST: 0.5, SM: 0.5 and ECC, BFlh: 0.8, ST: 1.0, SM: 0.8. One-sample *t*-tests were used to examine whether SWV changes were different from zero. Differences in average torque (absolute and relative) during the task and SWV changes among contraction types and muscles targeted during the elastography measurements were examined with separate two-way repeated-measures ANOVAs [contraction type (3) x muscle (3)]. T2 responses were examined with separate three-way repeated-measures ANOVAs [contraction type (3) × muscle (3) × time (3)]. Significant main effects and interactions were followed with post hoc pairwise comparisons with Bonferroni correction. For all ANOVAs, the assumption of sphericity was examined with Mauchly’s test and, when violated, the Greenhouse–Geisser correction was applied. Hedges’ g_av_ effect size was calculated for the *t*-tests and pairwise comparisons, and partial eta squared (η_p_^2^) for the ANOVAs (Lakens 2013). Bivariate correlations were examined with Pearson product-moment correlation coefficient. Statistical significance was set at *p* ≤ 0.05. SPSS v.25 (IBM Corp., Armonk, NY, USA) was used for all statistical analyses. The effect sizes were calculated with the spreadsheet provided in Lakens (2013).

## Results

### Torque

**Fig. 3:**
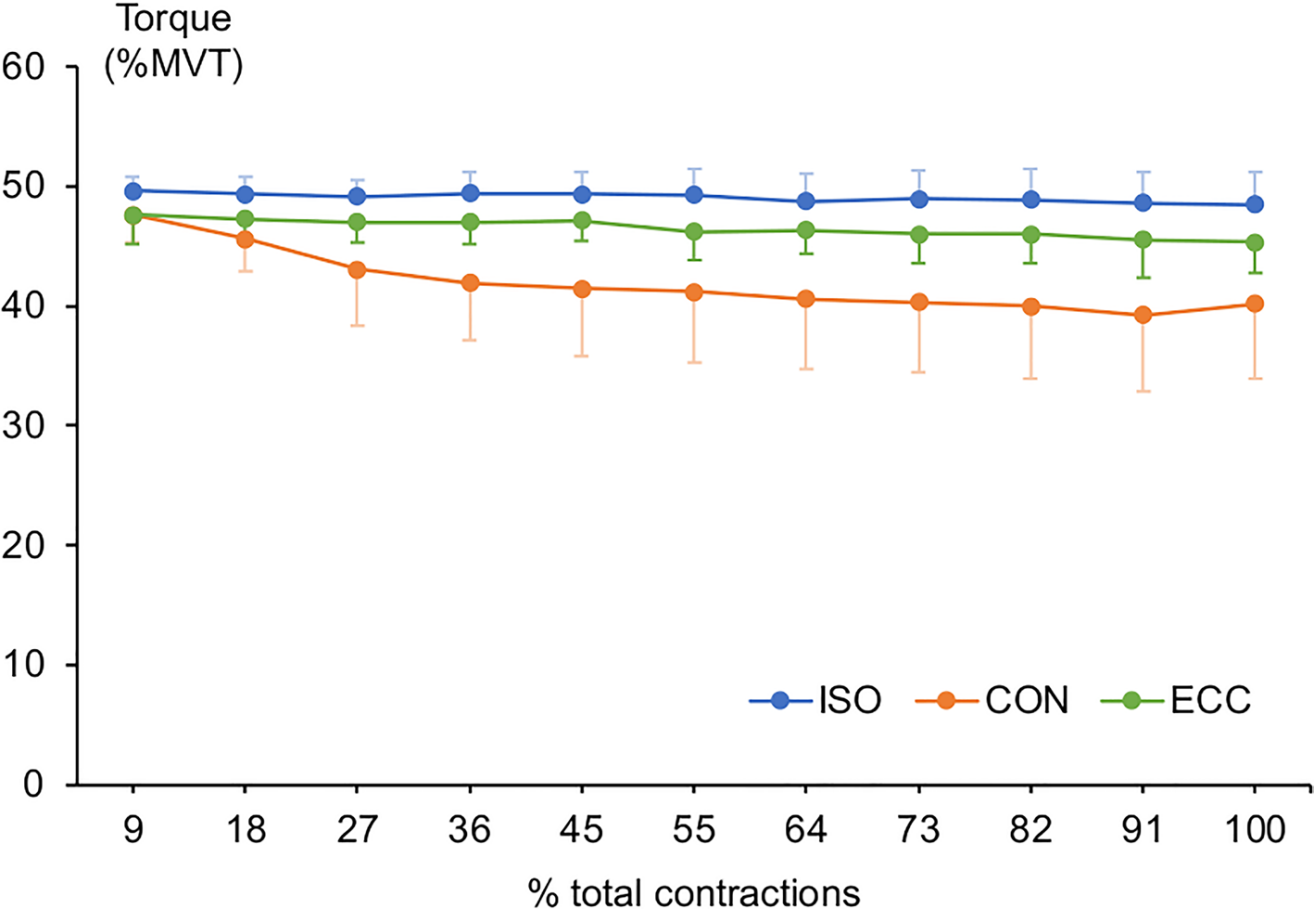
Time course of the knee flexor torque response during the fatiguing task in the isometric (ISO), concentric (CON), and eccentric (ECC) conditions. Data are presented as mean ± SD (*n* = 9)

In all conditions, MVT decreased after the fatiguing task (main effect of time, *F*_1,8_ = 43.61, *p* < 0.001, η_p_^2^ = 0.85; ISO [pre to post], 104.7 ± 20.7 to 84.2 ± 18.5 Nm, − 18.4%; CON, 118.9 ± 25.9 to 77.2 ± 15.9 Nm, − 33.6%; ECC, 109.4 ± 22.3 to 75.5 ± 17.8 Nm, − 30.9%) (Fig. 3), while there was a significant contraction type x time interaction (*F*_2,16_ = 3.733, *p* = 0.047, η_p_^2^ = 0.32) but no significant pairwise comparisons.

There was a significant main effect of contraction type on the average torque (normalised to MVT) exerted during the task (*F*_1.26,10.09_ = 15.862, *p* = 0.002, η_p_^2^ = 0.67), but no main effects of muscle targeted during the elastography measurements (*F*_2,14.35_ = 3.511, *p* = 0.054, η_p_^2^ = 0.31) or interaction (*p* = 0.413, η_p_^2^ = 0.11). Pairwise comparisons showed that participants maintained higher torque in ISO (49.1 ± 1.8%MVT) compared to CON (41.9 ± 4.7%MVT, *p* = 0.008, g_av_ = 1.81) and ECC (46.5 ± 2.0%MVT, *p* = 0.035, *g*_av_ = 1.22). Similarly, average torque in ECC was higher than CON (*p* = 0.018, *g*_av_ = 1.15). In absolute terms, however, the average torque exerted during the task exhibited no main effects of contraction type (*p* = 0.655) or muscle targeted during the elastography measurements (*p* = 0.113) and no interaction (*p* = 0.389). Collapsed across muscles, absolute torque was 51.3 ± 9.9 Nm for ISO, 49.3 ± 7.9 Nm for CON and 50.9 ± 10.1 Nm for ECC.

### Shear wave velocity (SWV)

**Fig. 4:**
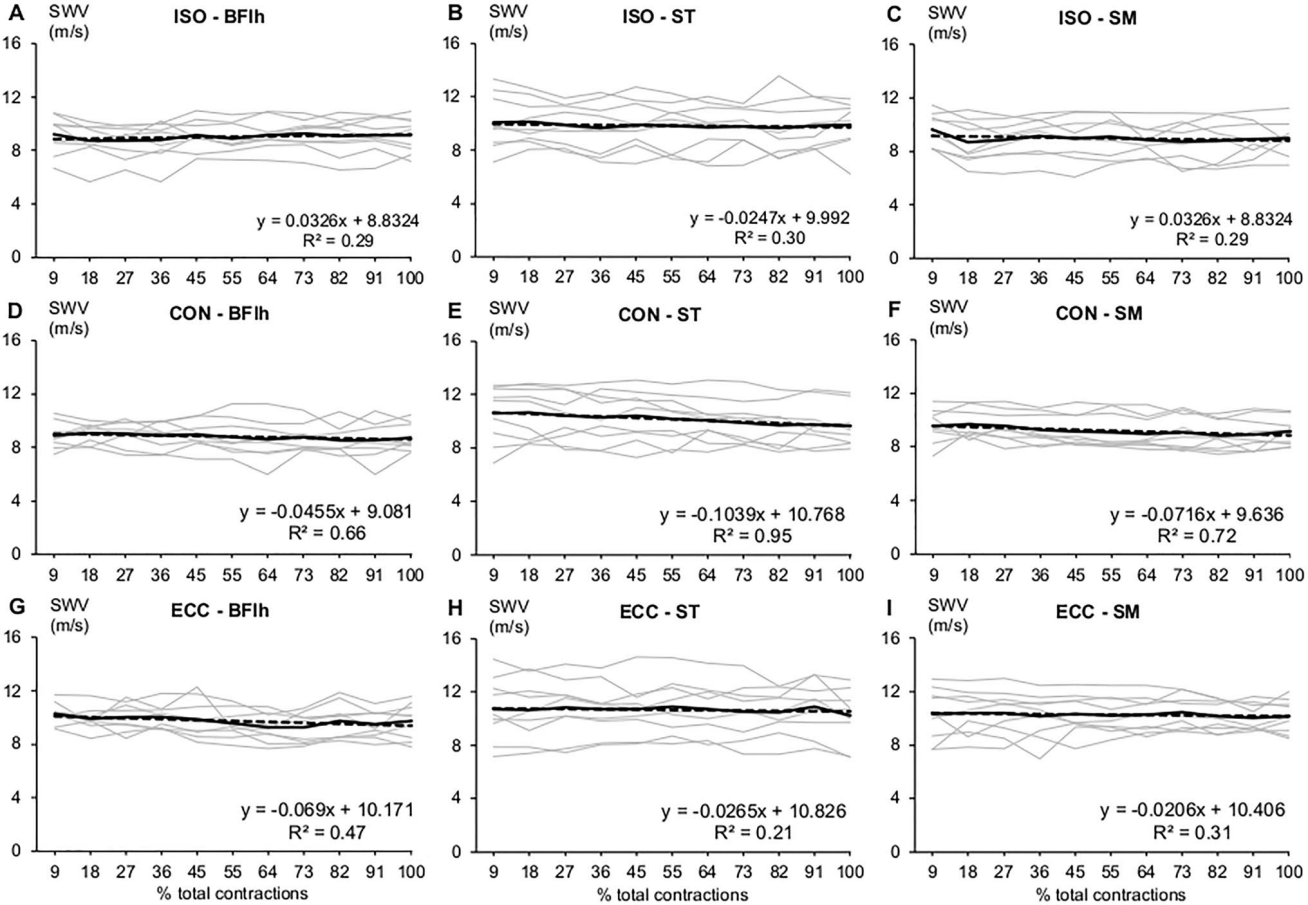
Hamstrings shear wave velocity (SWV) during the fatiguing task across all conditions. The grey lines are individual responses, the black solid line is the group mean SWV, and the black dashed line is the regression model fitted to the average data. *ISO* isometric, *CON* concentric, *ECC* eccentric. *BFlh* biceps femoris long head, *ST* semitendinosus, *SM* semimembranosus

On an individual level, muscle SWV responses during the task were highly variable in magnitude and direction, across muscles and conditions (Fig. 4, Online Resource 1). SWV changes reflected this variability especially in CON and ECC (Table 1). However, BFlh SWV response was more uniform across participants in ISO and ECC, albeit in opposite directions. In ISO, BFlh SWV increased in most participants (5/9), while ST and SM SWV was largely unchanged or decreased. The largest changes were seen in CON, where most participants exhibited decreased SWV in ST (5/9) and SM (6/9). In contrast, BFlh SWV remained consistent in most participants (5/9). Finally, in ECC, BFlh SWV either decreased (4/9) or remained unchanged (5/9), while that of ST was largely unchanged (7/9). In contrast, SM SWV responses were highly variable.

**Table 1:**
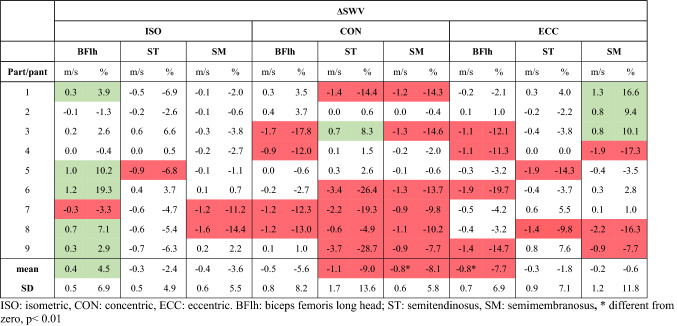
Individual and group (*n* = 9) shear wave velocity changes (ΔSWV) during the fatiguing task in all muscles and contraction types, in absolute and relative terms

Absolute changes were classified using the respective typical error (TE) of previous SWV measurements, within the same muscle and contraction type at 50% MVT, from the same cohort in fresh state (Evangelidis et al. 2021). Changes equal or higher/lower than ± TE were classified as increase (green)/decrease (red), respectively. Changes within that range were considered as non-substantial. For reference, the TE (m/s) used as a threshold for each muscle and contraction type was: ISO, BFlh: 0.3, ST: 0.8, SM: 1.0; CON, BFlh: 0.6, ST: 0.5, SM: 0.5 and ECC, BFlh: 0.8, ST: 1.0, SM: 0.8

On a group level, absolute SWV changes were significantly different from zero in SM CON (mean difference − 0.79 m/s, 95% CI − 1.20 to − 0.37, *p* = 0.003, *g*_av_ = 1.84), and in BFlh ECC (mean diff − 0.76 m/s, 95% CI − 1.27 to − 0.25, *p* = 0.009, *g*_av_ = 1.47), while there was an increasing trend in BFlh ISO (mean diff 0.35 m/s, 95%CI − 0.03 to –0.74, *p* = 0.064, *g*_av_ = 0.92).

A two-way repeated-measures ANOVA revealed a significant main effect of contraction type on SWV change (*F*_2,16_ = 3.678, *p* = 0.049, η_p_^2^ = 0.32); however, pairwise comparisons were not significant. Also, there was no main effect of muscle (*p* = 0.485) or contraction type × muscle interaction (*p* = 0.226).

#### Relationships between SWV and torque during the fatiguing task

**Fig. 5:**
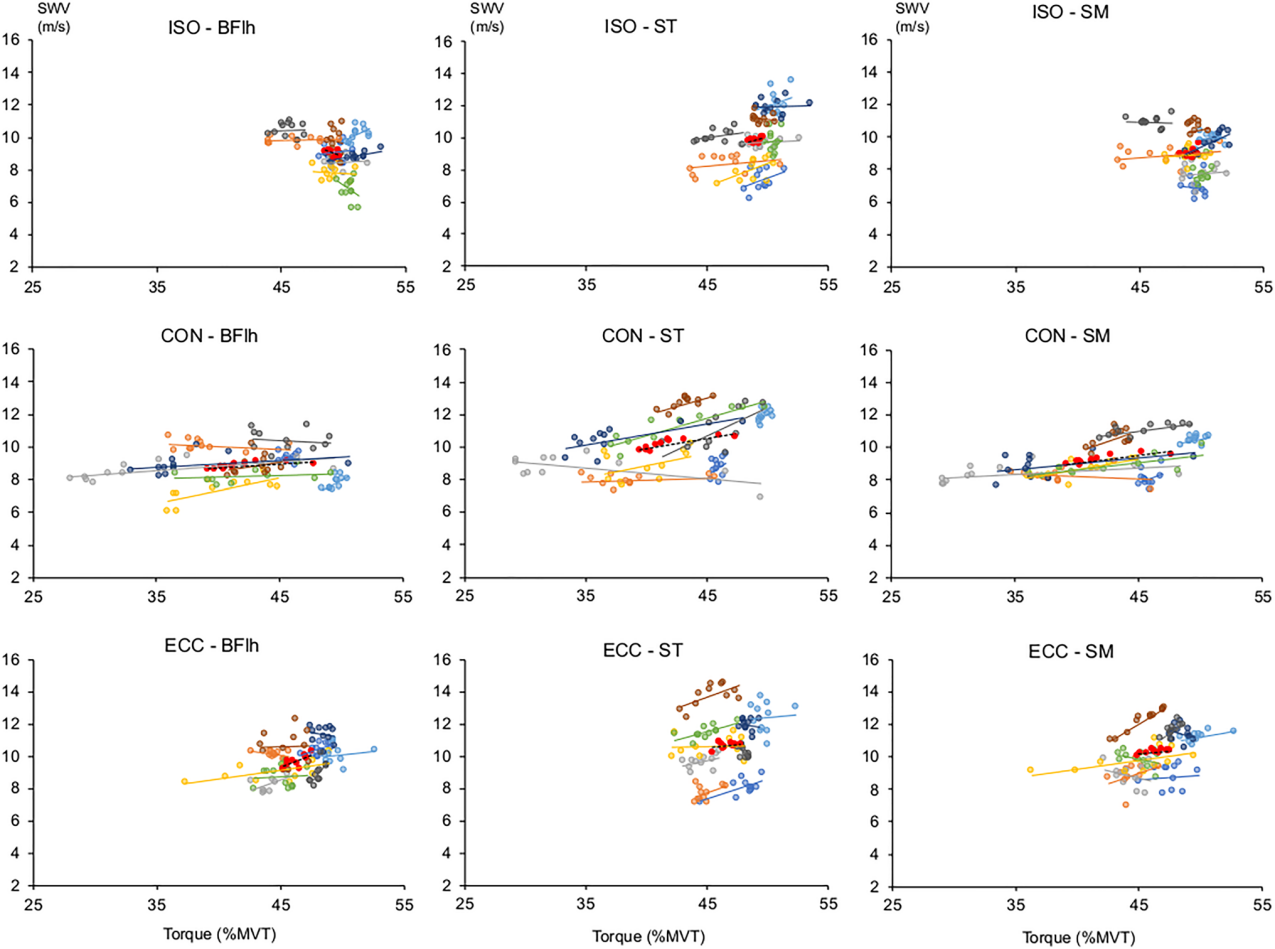
Bivariate correlations between individual and group (red circles, n = 9) knee flexion torque and hamstrings shear wave velocity (SWV) data during the fatiguing task across all conditions. Pearson’s r and significance level for all correlations are presented in Table 2. *ISO* isometric, *CON* concentric, *ECC* eccentric. *BFlh* biceps femoris long head, *ST* semitendinosus, *SM* semimembranosus

In all muscles, SWV and torque during the CON task were strongly related (Fig. 5, Table 2). Similarly, significant moderate-to-strong correlations were found for BFlh and SM in ECC, but only moderate correlations in ISO (*p* = 0.059–0.26).

**Table 2:** Pearson correlation coefficients (r) for the correlations between shear wave velocity (SWV) and relative torque (%MVT) exerted during the task

	ISO						CON						ECC					
	BFlh		ST		SM		BFlh		ST		SM		BFlh		ST		SM	
Part/pant	*r*	*p*	*r*	*p*	*r*	*p*	*r*	*p*	*r*	*p*	*r*	*p*	*r*	*p*	*r*	*p*	*r*	*p*
1	0.175	0.608	0.396	0.227	− 0.092	0.787	− 0.085	0.803	0.343	0.302	0.353	0.287	0.543	0.085	**0.660**	**0.027**	0.077	0.823
2	0.182	0.592	0.273	0.417	0.286	0.394	− 0.251	0.456	0.266	0.430	− 0.226	0.505	− 0.301	0.369	0.444	0.172	0.367	0.267
3	0.170	0.617	0.160	0.639	0.127	0.711	**0.625**	**0.040**	** − 0.611**	**0.046**	0.486	0.129	0.528	0.095	0.439	0.176	− 0.269	0.424
4	− 0.114	0.740	0.588	0.057	0.112	0.744	**0.730**	**0.011**	0.463	0.151	**0.700**	**0.017**	0.575	0.064	0.059	0.862	0.553	0.077
5	0.522	0.100	0.250	0.458	0.056	0.871	**0.658**	**0.028**	0.464	0.151	0.334	0.316	0.105	0.759	0.066	0.847	0.597	0.052
6	− 0.338	0.310	0.026	0.940	0.241	0.475	0.201	0.554	**0.910**	**0.000**	**0.723**	**0.012**	0.069	0.839	**0.681**	**0.021**	− 0.348	0.295
7	**0.609**	**0.047**	0.137	0.688	**0.684**	**0.020**	0.330	0.322	**0.701**	**0.016**	0.454	0.161	− 0.151	0.658	− 0.106	0.755	− 0.265	0.431
8	− 0.115	0.737	− 0.218	0.519	0.095	0.780	0.276	0.411	**0.715**	**0.013**	0.588	0.057	0.033	0.923	**0.631**	**0.037**	**0.962**	**0.000**
9	− 0.021	0.950	0.328	0.325	− 0.058	0.867	− 0.158	0.644	**0.687**	**0.019**	**0.767**	**0.006**	**0.708**	**0.015**	**− 0.626**	**0.039**	**0.713**	**0.014**
Group	− 0.584	0.059	0.514	0.105	0.575	0.064	**0.700**	**0.016**	**0.859**	**0.001**	**0.857**	**0.001**	**0.748**	**0.008**	0.351	0.290	**0.670**	**0.024**

Significant correlations (*p* ≤ 0.05) are denoted by bold font

*ISO* isometric, *CON* concentric, *ECC* eccentric. *BFlh* biceps femoris long head, *ST* semitendinosus, *SM* semimembranosus

#### Relationships between SWV and MVT changes

SWV changes were significantly correlated with knee flexor MVT changes only in SM in the CON condition (*r* = 0.74, *p* = 0.021). The respective correlations in all other muscles and conditions were non-significant (ISO, *r* = − 0.52 to 0.33, *p* = 0.147–0.732; CON, *r* = 0.23 to 0.26, *p* = 0.505–0.559; ECC, *r* = − 0.39 to 0.03, *p* = 0.338–0.948).

### T2 MRI

Table 3 presents the T2 values before and after the task for all muscles and conditions. There was a significant main effect of time (*F*_2,16_ = 25.253, *p* < 0.001. η_p_^2^ = 0.76), but not of muscle (*F*_2,16_ = 3.274, *p* = 0.064, η_p_^2^ = 0.29) or contraction type (*F*_2,16_ = 1.617, *p* = 0.229, η_p_^2^ = 0.17) and no interactions (*p* ≥ 0.183). Pairwise comparisons showed that T2 values increased after the task (*p* = 0.002) and returned to baseline 48 h later.

**Table 3:** Hamstrings T2 values (mean ± SD) before and after the fatiguing task in all conditions

		T2 transverse relaxation time (ms)		
		Pre	Post 0 h	Post 48 h
ISO	BFlh	38.5 ± 3.4	40.4 ± 3.3	38.0 ± 2.7
	ST	38.1 ± 3.6	40.8 ± 2.8	37.4 ± 3.1
	SM	37.6 ± 2.7	39.5 ± 2.6	37.3 ± 2.7
CON	BFlh	39.0 ± 3.2	42.0 ± 3.9	38.7 ± 3.8
	ST	38.5 ± 3.8	42.5 ± 2.7	37.8 ± 3.7
	SM	38.2 ± 3.1	42.2 ± 3.5	38.5 ± 3.7
ECC	BFlh	40.4 ± 1.7	42.2 ± 2.0	40.8 ± 3.1
	ST	40.2 ± 2.2	41.7 ± 2.2	40.2 ± 2.8
	SM	39.5 ± 1.8	41.6 ± 2.5	40.2 ± 2.6

*ISO* isometric, *CON* concentric, *ECC* eccentric. *BFlh* biceps femoris long head, *ST* semitendinosus, *SM* semimembranosus

#### Relationships between T2 and MVT changes

**Fig. 6:**
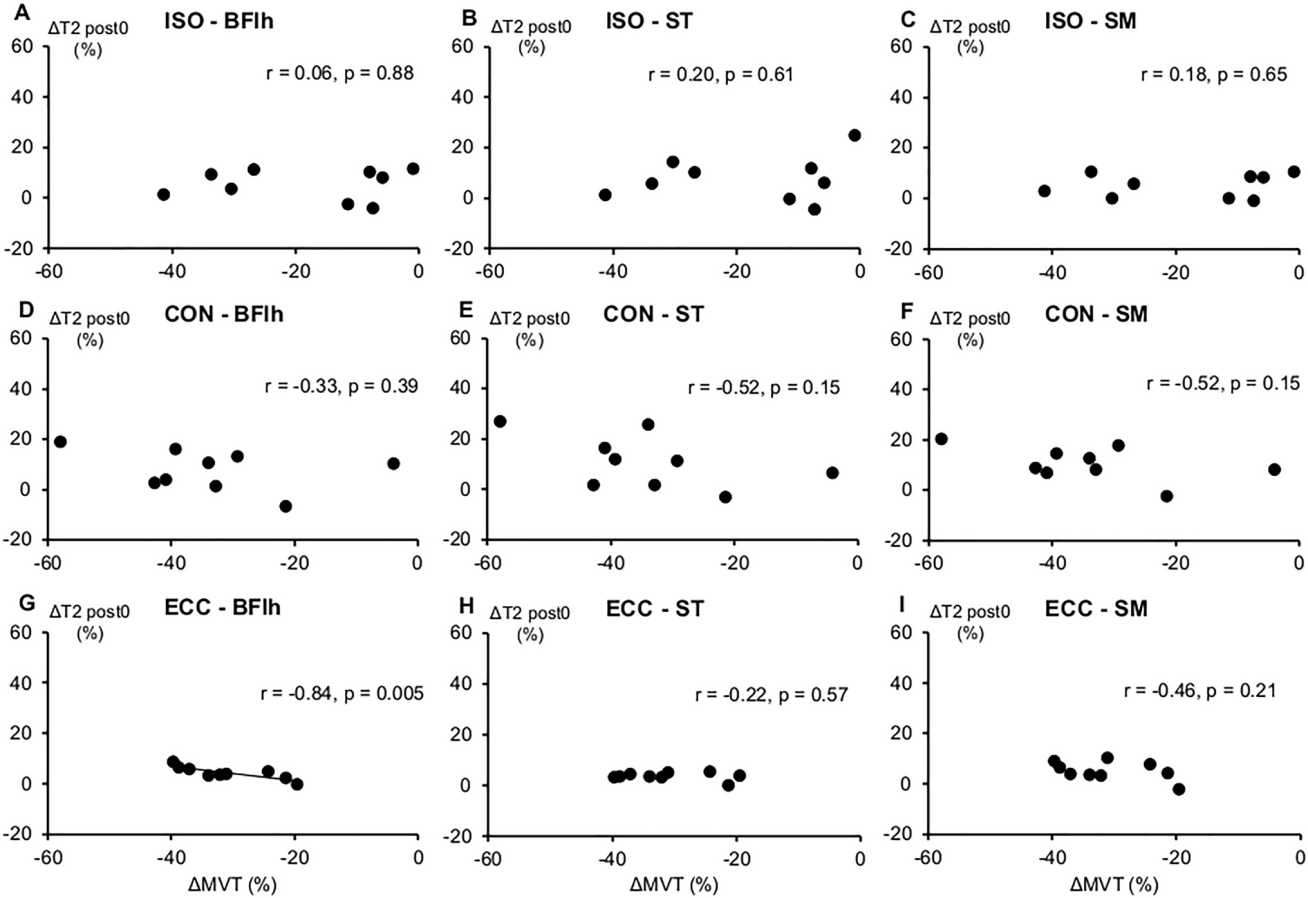
Bivariate correlations between T2 values and knee flexion maximal voluntary torque (MVT) changes after the fatiguing task across conditions and hamstring muscles. Each dot represents one participant (*n* = 9). *ISO* isometric, *CON* concentric, *ECC* eccentric. *BFlh* biceps femoris long head, *ST* semitendinosus, *SM* semimembranosus

BFlh T2 post 0 h and MVT changes exhibited a strong, inverse relationship in ECC (*r* = − 0.84, *p* = 0.005) (Fig. 6). Moderate but non-significant correlations between ΔT2 and ΔMVT were also found for ST and SM in CON, while no correlation was found in ISO. No significant correlations were found between T2 post 48 h and MVT changes in any condition.

#### Relationships between T2 and SWV changes

No significant correlations were found between T2 and SWV changes for any muscle or condition either immediately after the task or 48 h later, with the exception of SM immediately after the CON task (Table 4).

**Table 4:** Pearson correlation coefficients for the correlations between T2 and SWV changes across the hamstring muscles and contraction types

		Post 0 h		Post 48 h	
		*r*	*p*	*r*	*p*
ISO	BFlh	0.20	0.616	0.09	0.818
	ST	0.04	0.917	0.43	0.245
	SM	0.07	0.849	0.13	0.739
CON	BFlh	− 0.37	0.324	− 0.15	0.704
	ST	− 0.21	0.595	0.03	0.943
	SM	− 0.67	0.047	− 0.37	0.323
ECC	BFlh	0.19	0.633	0.50	0.168
	ST	− 0.37	0.325	0.35	0.364
	SM	0.36	0.338	0.65	0.060

*ISO* isometric, *CON* concentric, *ECC* eccentric. *BFlh* biceps femoris long head, *ST* semitendinosus, *SM* semimembranosus

## Discussion

The aim of this study was to investigate (1) the effect of fatigue on hamstrings active muscle stiffness and load bearing, and (2) whether any effects are specific to contraction type, using shear wave elastography and MRI. First, any comparison of the SWV responses among conditions should be made with caution as the average relative torque (%MVT) sustained during the fatigue task was different. Nevertheless, the average absolute torque (and thus the mechanical output) was similar among conditions. In partial confirmation of our hypotheses, we found that BFlh SWV decreased in ECC, but exhibited an increasing trend in ISO. In contrast, ST SWV decreased in CON, while it was relatively unchanged in ISO and ECC. Finally, SM SWV largely decreased in CON, but its response was highly variable in ECC with evidence of increased load bearing. At the group level, SWV responses were accounted for, in large part, by torque changes during the CON task, but to a lesser extent in ISO and ECC, although the relationship between knee flexor torque and SWV responses during the task was less clear at the individual level. The opposite direction of SWV changes among the hamstring muscles and contraction types suggests that fatigue causes a load redistribution within this group, depending upon contraction type. Interestingly, BFlh active stiffness during fatiguing eccentric contractions was more compromised than ST and SM active stiffness, which may provide an insight into the muscle’s susceptibility to strain injuries and highlights the role of fatigue as an important risk factor.

Previous studies suggested that fatigue did not affect all individual hamstring muscles equally; however, their findings were conflicting potentially due to the different fatiguing tasks and contraction types examined (Timmins et al. 2014; Marshall et al. 2014; Mendes et al. 2020; Freitas et al. 2021). Our results from direct measurement of local active muscle stiffness using SWE support the above notion. While the high interindividual variability found in our study has likely blunted the magnitude of the group changes, a closer inspection of the data reveals some systematic trends. We found that BFlh active stiffness decreased in ECC, in line with previous evidence that the myoelectrical activity of the BFlh decreased, while it remained unchanged for the medial hamstrings following repeated sprints or during a simulated soccer match (Timmins et al. 2014; Marshall et al. 2014). Active muscle stiffness is closely related to EMG activity and torque (Nordez and Hug 2010; Ateş et al. 2015; Barron et al. 2022) and, at the fibre level, with the number of attached cross-bridges (Ford et al. 1981), thereby our results could partly reflect the reduced BFlh force production due to fatigue. In contrast, active muscle stiffness in ST and SM was largely unchanged or increased in most participants, indicating a degree of load redistribution from the fatigued BFlh to the less-affected medial hamstrings. The notion that BFlh was more fatigued than ST and SM during ECC is further corroborated by the fact that: (1) the BFlh T2 increase immediately after task completion [an index of metabolic activity and recruitment (Foley et al. 1999)] accounted for 70% of the MVT decrease, while ST and SM T2 changes exhibited only low-to-moderate non-significant correlations with MVT change, and (2) BFlh ECC SWV exhibited the strongest association with knee flexor torque during the fatiguing task compared to the other hamstring muscles (*r* = 0.75). Collectively, these observations suggest that fatiguing eccentric contractions mostly compromise BFlh mechanical properties and load bearing capacity, while this is partly compensated by increased contribution from the medial hamstrings.

The greater decrease in BFlh active stiffness in ECC (compared to ST and SM) found in this study may reflect greater muscle damage in BFlh than the medial hamstring muscles. Modelling studies suggest that, during eccentric muscle actions of sprint running, BFlh sustains the largest strains among all individual hamstring muscles (Chumanov et al. 2011; Schache et al. 2012). Considering that muscle damage is determined by the magnitude of fibre active strain (Lieber and Friden 1993), it is likely that eccentric contractions cause greater muscle damage to BFlh compared to ST and SM. Yet, the limited data on the length-tension relationship of the individual hamstring muscles do not provide clear support for this notion as they suggest that all three biarticular hamstring muscles operate at the descending limb of their length–tension curve at the hip and knee joint angles examined in this study (Cutts 1988; Chleboun et al. 2001). Delayed (> 1 day) increases in muscle T2 relaxation times following eccentric exercise are considered to reflect muscle damage (Foley et al. 1999); however, there were only moderate non-significant correlations between SWV and post 48 h T2 changes in BFlh (*r* = 0.50) and SM (*r* = 0.65).

Muscle damage following eccentric contractions is greater in type II fibres (Fridén et al. 1983). Although data on hamstrings muscle composition are scarce, they show a relatively balanced and comparable fibre-type distribution across its constituent muscles (Garrett et al. 1984; Shalabi et al. 2002; Dahmane et al. 2006; Evangelidis et al. 2017). Type II-dominant muscles exhibit greater reduction in their force-generating capacity with fatigue (Tesch 1980; Hamada et al. 2003) which could in turn be reflected in their active stiffness. However, we did not find any significant correlation between knee flexor MVT and SWV changes (except for SM in CON). Therefore, differences in fibre type are not likely to explain the diverse active stiffness responses across the hamstrings seen here. Nevertheless, the high interindividual variability in hamstrings fibre-type composition suggests that individuals with high percentage of type II muscle fibres may exhibit more pronounced decrements in muscle force and active stiffness with fatigue. Clearly, this speculation remains to be examined directly.

These findings may provide some insight into the high injury rates in BFlh. BFlh strain injuries typically occur during high-speed running, presumably during the late-swing phase (Heiderscheit et al. 2005; Schache et al. 2009), when hamstrings undergo large eccentric loading and BFlh sustains the largest strains among hamstrings (Schache et al. 2012). In fresh state, BFlh is more likely to withstand these loads due to the high forces developed by its contractile and passive elastic components, which can resist the resultant strains and absorb the energy successfully. However, as fatigue ensues, BFlh force-generating capacity and active stiffness decrease, and muscle fascicles become less capable to resist the strains, potentially leading to muscle injury (Mair et al. 1996). Some participants exhibited increased and/or constant SM and ST active stiffness in ECC, suggesting an increased relative load borne by the medial hamstrings, potentially reflecting a compensating mechanism for the decreasing BFlh contribution. However, such stiffness gradient among the hamstring muscles may exacerbate the strains experienced by BFlh during eccentric contractions. BFlh and ST are closely connected anatomically, sharing a proximal tendon (Woodley and Mercer 2005; van der Made et al. 2013). The decreasing stiffness of the fatigued BFlh, combined with the relatively higher stiffness of the ST, could result in large, localised shearing stresses and strains along their proximal myotendinous connections. While ST can theoretically better accommodate such overload, both mechanically (higher active stiffness) and structurally (longer fascicles), a fatigued BFlh with compromised active stiffness and shorter fascicles (Kellis et al. 2012) would be more exposed to excessive strains and subsequent injury.

In ISO, BFlh SWV increased or remained constant for all participants but one, while ST and SM SWV remained largely constant or decreased (but did not increase), indicating larger relative load borne by BFlh. These changes are opposite to those seen in ECC suggesting that fatigue affects hamstrings load bearing differently, depending on contraction type. A possible explanation may be that participants were able to better sustain the target torque in ISO than ECC, potentially due to lower levels of fatigue induced by isometric contractions. Nevertheless, the average absolute torque sustained, and therefore, the muscle mechanical output was similar between conditions.

The greater relative load borne by BFlh (compared to the medial hamstrings) during ISO is consistent with previous shear wave elastography studies (Mendes et al. 2020; Freitas et al. 2021). In those studies, BFlh active stiffness remained constant during a sustained isometric contraction at 20%MVC, while that of ST was reduced, thereby increasing the BFlh/ST ratio towards the end of the task (Mendes et al. 2020; Freitas et al. 2021). It is unclear why we found different ST responses compared to those studies; however, it may be due to the different fatiguing tasks employed. In our study, participants completed 99 submaximal intermittent isometric contractions (49.1%MVT, total time under contraction: ~ 495 s), while in the aforementioned studies, participants performed low-force sustained isometric contractions until exhaustion (20%MVC, duration: 399–475 s) (Mendes et al. 2020; Freitas et al. 2021). Sustained isometric contractions may induce fatigue faster than intermittent contractions (Sjøgaard et al. 1988). Indeed, although we employed a task with longer duration and higher intensity, knee flexor torque decreased only by 1.7% vs. 5% (the defined point of exhaustion) in the previous studies (Mendes et al. 2020; Freitas et al. 2021).

Nonetheless, ISO seems to increase BFlh load bearing relative to ST, potentially reflecting the ensuing fatigue and increased effort to maintain the target torque. The notion that BFlh recruitment increased is supported by the negative correlation between BFlh SWV and torque during the ISO task (*r* = − 0.58, *p* = 0.059), suggesting that, as participants became fatigued and exerted less torque, BFlh active stiffness tended to increase (greater BFlh load bearing). Yet, BFlh T2 changes were unrelated to MVT changes likely because, overall, ISO did not induce substantial fatigue in our participants, as evidenced by the well-maintained target torque throughout the task and the moderate reduction in MVT (− 18.4%).

The CON task caused the greatest fatigue in hamstrings, reflected in the largest MVT decrease across all contraction types (− 33.6%) and the lowest average relative torque sustained during the task (41.9%MVT). This is in accordance with the higher muscle activation and metabolic cost expected in concentric vs. isometric and eccentric contractions (Bigland-Ritchie and Woods 1976). Similarly, ST and SM SWV exhibited up to a fourfold greater decrease in CON compared to the other conditions, although these may partly reflect the lower torque sustained during the CON task. Notably, on a group level, BFlh SWV exhibited the smallest decrease in CON, which is in direct contrast to its response in ISO and ECC (discussed above). This pattern of changes suggests that, in concentric tasks, ST and SM are more fatigued than BFlh. This notion is further supported by the higher correlations between SWV and torque (during the task) in ST and SM (both *r* = 0.86, *p* < 0.001) than in BFlh (*r* = 0.70, *p* = 0.016). Similarly, the correlations between T2 (post 0 h) and MVT changes following CON were higher in ST and SM (both *r* = − 0.52) than in BFlh (*r* = − 0.33), although none was statistically significant. This pattern of hamstrings active stiffness is in line with our previous study in fresh state (Evangelidis et al. 2021), suggesting that fatigue during concentric contractions does not alter the pattern of load distribution within hamstrings, in contrast to eccentric contractions.

This study has several limitations. First, constraints in time and resources limited the number of recruited participants. For this reason, we focused on a more qualitative interpretation of the data. Further examination of larger cohorts is clearly needed to confirm our findings. Second, the intensity of the fatiguing task was moderate, and this may have affected the magnitude of the SWV changes. As fatigue causes smaller changes in muscle stiffness than force (Edman and Lou 1990), higher exercise intensity may have resulted in more pronounced SWV responses. However, we opted to use 50%MVC contractions to avoid any signal saturation in the SWV measurements. Third, the average relative torque (%MVT) exerted during the fatiguing task was different among conditions and any comparison should be made with caution. Nevertheless, the average absolute torque, and therefore the mechanical output, was similar in all conditions. Fourth, we examined the SWV changes in the three largest hamstring muscles; however, other muscles also contribute to knee flexion torque and load bearing (biceps femoris short head, sartorius, gracilis, and gastrocnemius) which may have contributed to the lack of clear evidence of load redistribution in our study. Finally, we did not measure muscle temperature, which can directly affect active SWV. Increased muscle temperature causes a reduction in active stiffness that is independent of muscle force (Bernabei et al. 2020), confounding any inferences regarding load bearing changes. Muscle temperature would be expected to primarily affect the responses in the CON task, due to its higher metabolic cost. However, in all muscles, most of the variance in CON SWV responses was explained by torque (49–74%); therefore, any potential impact of muscle temperature would have been rather limited. In contrast, eccentric contractions have the lowest metabolic cost, and therefore, muscle temperature is not expected to be significantly affected. Similarly, in ISO, SWV remained largely constant for all muscles with an increasing trend for BFlh, again suggesting a minimal, if any, effect of muscle temperature on our results.

## Conclusions

In conclusion, we found preliminary evidence of differential fatigue within the hamstrings that appears to be affected by contraction type. Interestingly, fatigue induced by eccentric contractions primarily compromises BFlh mechanical properties and load bearing capacity, which may explain the high rate of strain injuries sustained by this muscle. In contrast, BFlh seems to be the least fatigued hamstring muscle during an isometric task. Finally, concentric knee flexions induce the most pronounced mechanical and metabolic activity changes of all contraction types, primarily affecting the medial hamstrings (ST and SM).

## Supplementary Information

Below is the link to the electronic supplementary material.

ESM 1 Individual shear wave velocity (SWV, solid lines) and torque (T, dashed lines) responses for the biceps femoris long head (BFlh), semitendinosus (ST) and semimembranosus (SM) muscles during a fatiguing task of 99 submaximal intermittent isometric (ISO, panel A), concentric (CON, panel B) and eccentric (ECC, panel C) contractions (PDF 655 KB)

Supplementary file2 (XLSX 105 KB)

## Data Availability

All data generated or analysed during this study are included in this published article (and its supplementary information files).

## Abbreviations

ANOVA: Analysis of variance
BFlh: Biceps femoris long head
CON: Concentric condition
ECC: Eccentric condition
EMG: Electromyography
HSI: Hamstring strain injury
ISO: Isometric condition
MRI: Magnetic resonance imaging
MVC: Maximal voluntary contraction
MVT: Maximal voluntary torque
ROI: Region of interest
SM: Semimembranosus
ST: Semitendinosus
SWE: Shear wave elastography
SWV: Shear wave velocity
TE: Typical error
