# Supplementary material for: Fatigue-induced changes in hamstrings’ active muscle stiffness: effect of contraction type and implications for strain injuries
Source: Eur J Appl Physiol. 2022 Dec 10;123(4):833–46. doi: 10.1007/s00421-022-05104-0 (PMC10030419; doi:10.1007/s00421-022-05104-0)
Supplement: Supplementary file 1 — ESM 1 Individual shear wave velocity (SWV, solid lines) and torque (T, dashed lines) responses for the biceps femoris long head (BFlh), semitendinosus (ST) and semimembranosus (SM) muscles during a fatiguing task of 99 submaximal intermittent isometric (ISO, panel A), concentric (CON, panel B) and eccentric (ECC, panel C) contractions (PDF 655 KB) [file 421_2022_5104_MOESM1_ESM.pdf]

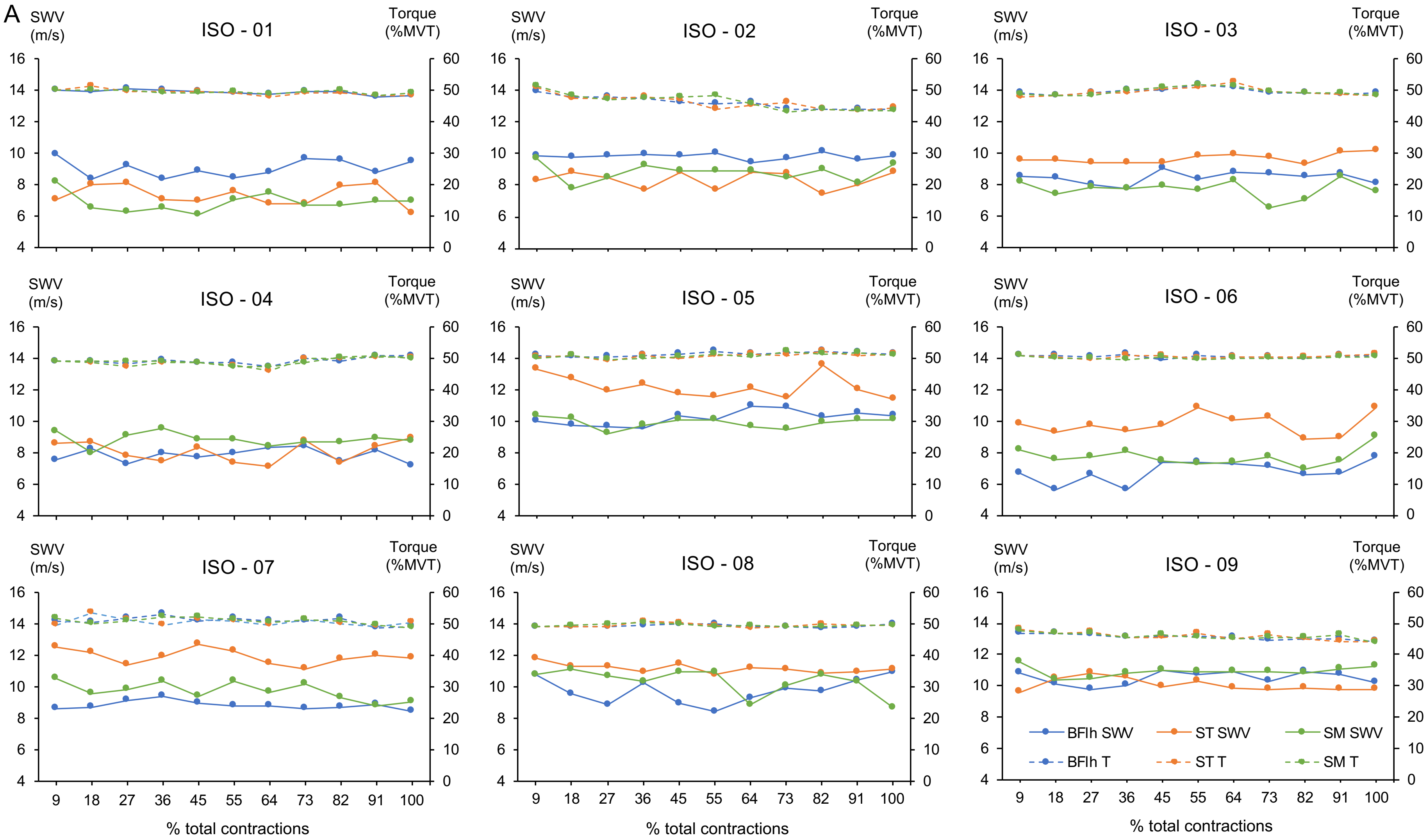
A
SWV (m/s)
Torque (%MVT)
ISO - 01
ISO - 02
ISO - 03
ISO - 04
ISO - 05
ISO - 06
ISO - 07
ISO - 08
ISO - 09
% total contractions
BFlh SWV
ST SWV
SM SWV
BFlh T
ST T
SM T

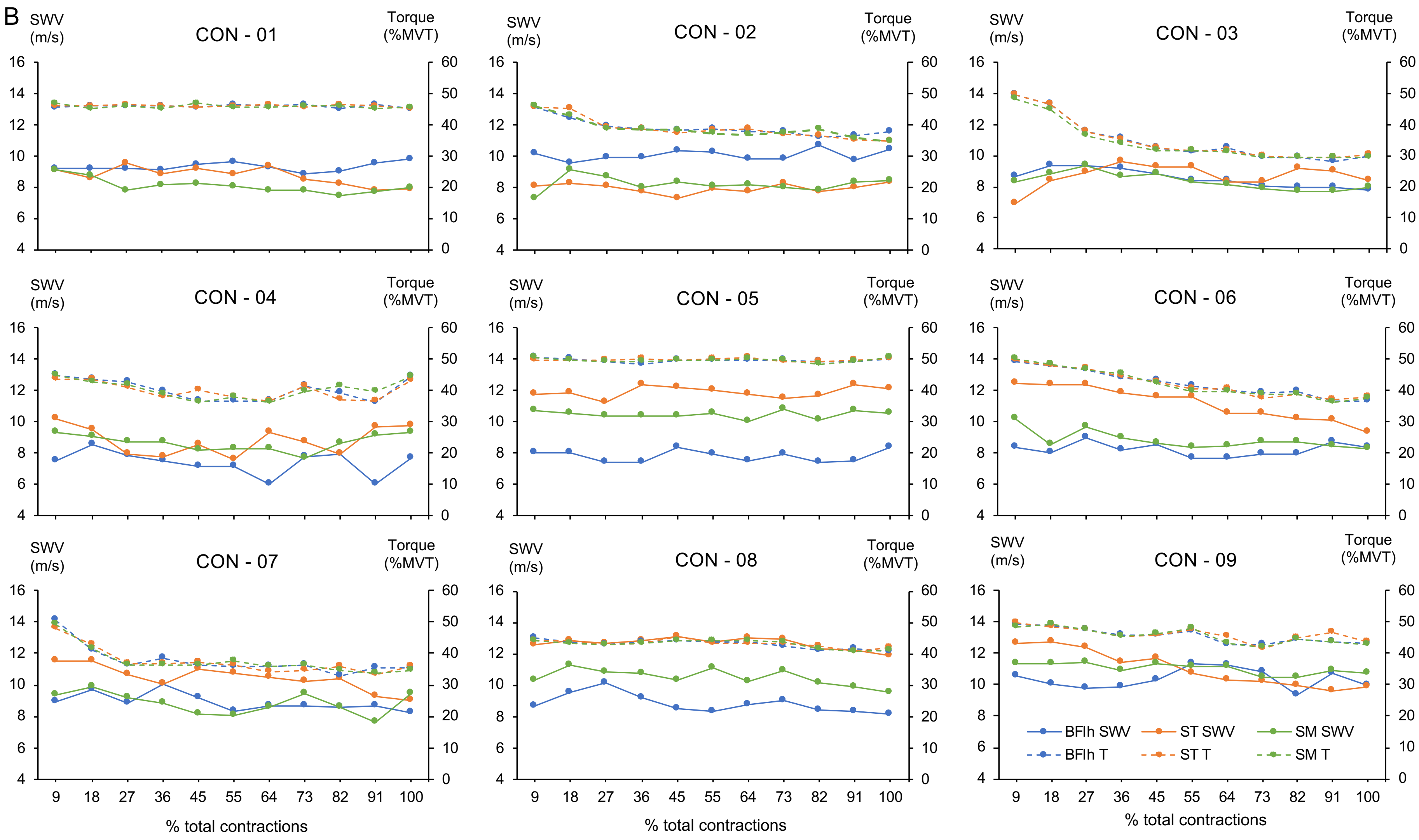
B
SWV (m/s)
Torque (%MVT)
CON - 01
CON - 02
CON - 03
CON - 04
CON - 05
CON - 06
CON - 07
CON - 08
CON - 09
16
14
12
10
8
6
4
60
50
40
30
20
10
0
9
18
27
36
45
55
64
73
82
91
100
% total contractions
BFlh SWV
ST SWV
SM SWV
BFlh T
ST T
SM T

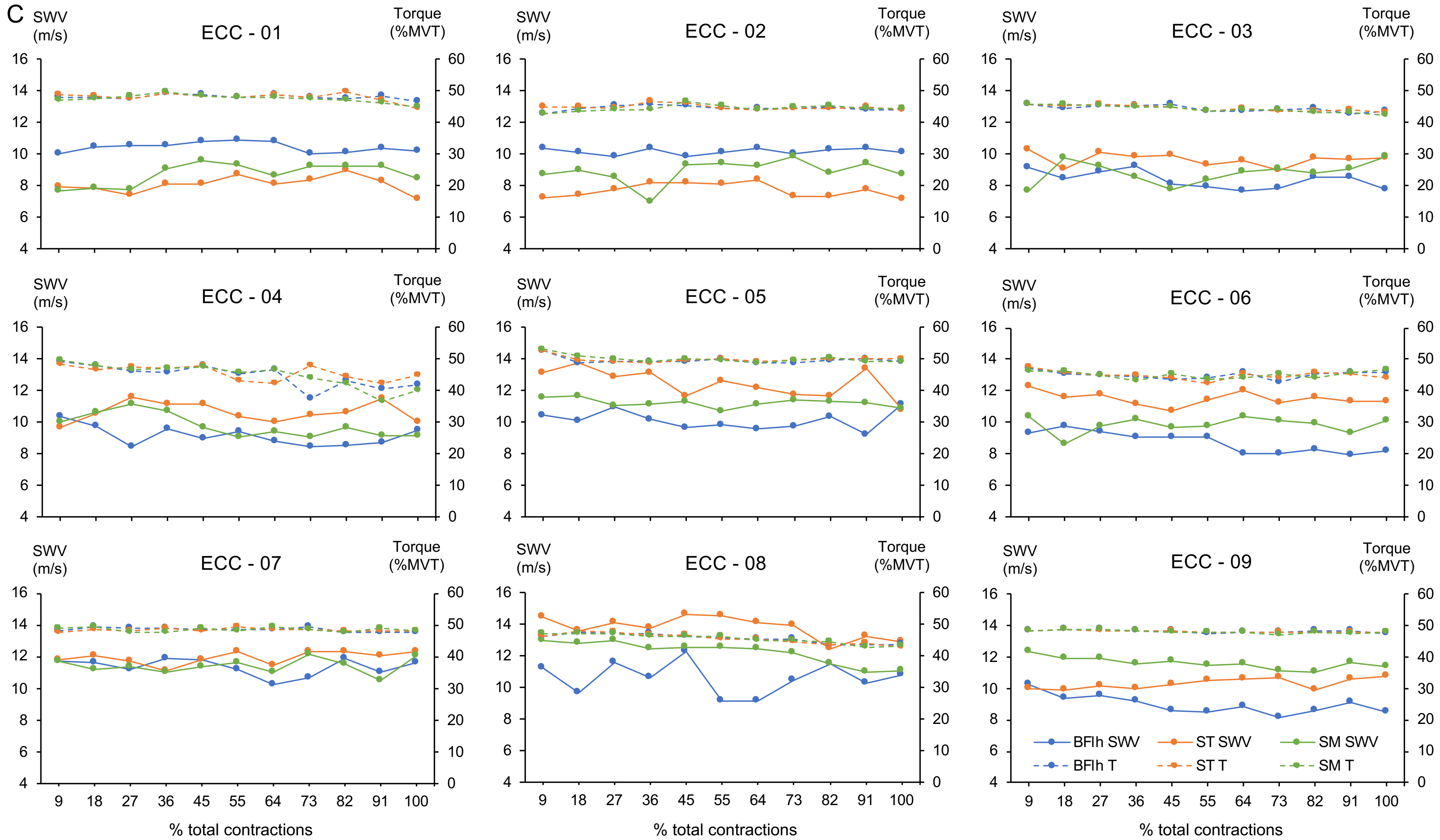

ESM 1 Individual shear wave velocity (SWV, solid lines) and torque (T, dashed lines) responses for biceps femoris long head (BFlh), semitendinosus (ST) and semimembranosus (SM) muscles during a fatiguing task of 99 submaximal intermittent isometric (ISO, panel A), concentric (CON, panel B) and eccentric (ECC, panel C) contractions.

Article title: Fatigue-induced changes in hamstrings' active muscle stiffness: Effect of contraction type and implications for strain injuries

Journal: European Journal of Applied Physiology

Authors: Pavlos E. Evangelidis[1,2], Xiyao Shan[3], Shun Otsuka[3], Chi Yang[4], Takaki Yamagishi[1], Yasuo Kawakami[1]

Affiliations: [1]Faculty of Sport Sciences, Waseda University, Japan, [2]Japan Society for the Promotion of Science, Japan, [3]Department of Anatomy, Aichi Medical University, Japan, [4]Graduate school of Sport Sciences, Waseda University, Japan

Corresponding author: Pavlos E. Evangelidis, pavlos.evangelidis@gmail.com
